# PARV4 prevalence, phylogeny, immunology and coinfection with HIV, HBV and HCV in a multicentre African cohort

**DOI:** 10.12688/wellcomeopenres.11135.1

**Published:** 2017-04-07

**Authors:** Colin P. Sharp, William F. Gregory, Louise Hattingh, Amna Malik, Emily Adland, Samantha Daniels, Anriette van Zyl, Jonathan M. Carlson, Susan Wareing, Anthony Ogwu, Roger Shapiro, Lynn Riddell, Fabian Chen, Thumbi Ndung'u, Philip J.R. Goulder, Paul Klenerman, Peter Simmonds, Pieter Jooste, Philippa C. Matthews

**Affiliations:** 1 Roslin Institute, University of Edinburgh, Edinburgh, EH25 9RG, UK; 2 Edinburgh Genomics, University of Edinburgh, Edinburgh, EH9 3FL, UK; 3 Kimberley Hospital, Kimberley, Northern Cape, 8301, South Africa; 4 Department of Paediatrics, University of Oxford, Oxford, OX1 3SY, UK; 5 Microsoft Research, Redmond, WA, 98052, USA; 6 Department of Microbiology and Infectious Diseases, Oxford University Hospitals NHS Foundation Trust, John Radcliffe Hospital, Oxford, OX3 9DU, UK; 7 Botswana Harvard AIDS Institute Partnership, Gaborone, Botswana; 8 Northampton General Hospital NHS Trust, Northampton, NN1 5BD, UK; 9 Royal Berkshire Hospital, Reading, RG1 5AN, UK; 10 HIV Pathogenesis Program, Nelson R. Mandela School of Medicine, University of KwaZulu-Natal, Durban, KwaZulu-Natal, 4041, South Africa; 11 Nuffield Department of Medicine, University of Oxford, Oxford, OX1 3SY, UK; 12 NIHR Biomedical Research Centre, John Radcliffe Hospital, Oxford, OX3 9DU, UK

**Keywords:** PARV4, parvovirus, Africa, co-infection, epidemiology, HBV, HCV, HIV

## Abstract

*Background: *The seroprevalence of human parvovirus-4 (PARV4) varies considerably by region. In sub-Saharan Africa, seroprevalence is high in the general population, but little is known about the transmission routes or the prevalence of coinfection with blood-borne viruses, HBV, HCV and HIV. *Methods: *To further explore the characteristics of PARV4 in this setting, with a particular focus on the prevalence and significance of coinfection, we screened a cohort of 695 individuals recruited from Durban and Kimberley (South Africa) and Gaborone (Botswana) for PARV4 IgG and DNA, as well as documenting HIV, HBV and HCV status. *Results: *Within these cohorts, 69% of subjects were HIV-positive. We identified no cases of HCV by PCR, but 7.4% were positive for HBsAg. PARV4 IgG was positive in 42%; seroprevalence was higher in adults (69%) compared to children (21%) (p<0.0001) and in HIV-positive (52%) compared to HIV-negative individuals (24%) (p<0.0001), but there was no association with HBsAg status. We developed an on-line tool to allow visualization of coinfection data ( https://purl.oclc.org/coinfection-viz). We identified five subjects who were PCR-positive for PARV4 genotype-3. *Ex vivo *CD8+ T cell responses spanned the entire PARV4 proteome and we propose a novel HLA-B*57:03-restricted epitope within the NS protein. *Conclusions: *This characterisation of PARV4 infection provides enhanced insights into the epidemiology of infection and co-infection in African cohorts, and provides the foundations for planning further focused studies to elucidate transmission pathways, immune responses, and the clinical significance of this organism.

**Abbreviations:** BBV, blood borne virus; HBV, Hepatitis B Virus; HBsAg, Hepatitis B surface antigen; HCV, Hepatitis C Virus; HIV, Human Immunodeficiency Virus; IgG, Immunoglobin G; PARV4, Human parvovirus 4; sSA, sub-Saharan Africa.

## Introduction

Human parvovirus-4 (‘PARV4’) is a single-stranded DNA virus in the family *Parvoviridae* ^1^. Its clinical significance remains uncertain ^2^, and epidemiology varies strikingly by region, for reasons that are not yet understood. The risk factors that operate in various settings appear to be very different. Studies of North American and European populations have reported that PARV4 exposure (IgG positive status) is strongly associated with parenteral risk factors, and with infection with blood-borne viruses (BBV’s), HIV, HCV and HBV ^3– 9^. An acceleration of HIV disease has also been described in association with PARV4 infection in a European cohort of HIV-infected subjects, although this effect may be confounded by the high prevalence of HCV co-infection ^6^. In contrast, in sub-Saharan Africa (sSA), serological evidence of PARV4 infection in the general population ranges from 4–37% and there is a paucity of data to support any consistent relationship with other BBVs ^10– 13^.

In a previous smaller study of mothers and children in South Africa (n=157), we found a high seroprevalence of PARV4 IgG (37%), but no cases of detectable viraemia, and demonstrated a relationship between older age and increasing PARV4 IgG prevalence ^11^. Despite the high population prevalence in sSA, little is known about the routes or risk factors for transmission, host immune responses, prevalence of viraemia, or clinical impact of PARV4 ^2, 14^.

The consistent evidence that PARV4 is endemic in populations in sSA prompted us to investigate further, using pre-existing cohorts to form a clearer view of the patterns of infection in these populations and to develop further insights into adaptive immune responses associated with PARV4 infection. Given previous evidence for the substantial influence of HLA Class I genotype on the outcome of viral infections (best exemplified in this population by our studies of HIV ^15, 16^ and HBV ^17^), we also set out to identify whether any such HLA-mediated effect can be observed with respect to PARV4 in the same cohorts. Previous work has demonstrated that high magnitude CD8+ T cell responses to PARV4 NS protein are maintained in the long-term ^7^; we expanded on this observation by screening PARV4 IgG-positive individuals for T cell responses spanning the entire PARV4 proteome.

Therefore, our specific aims in this expanded African cohort were as follows:

i To assimilate data for PARV4, HIV, HBV and HCV status from pre-existing cohorts and to describe the patterns of coinfection;
ii To seek any evidence of a relationship between positive PARV4 IgG status and acceleration of HIV disease;
iii To screen our study subjects for PARV4 viraemia in order to establish how prevalent this is, hypothesising that viraemia might be associated with age, pregnancy or HIV infection, and to derive sequences from viraemic subjects;
iv To investigate any significant impact of host HLA Class I genotype on PARV4 status and to improve *ex vivo* characterization of the CD8+ T cell response.

## Materials and methods

### Patient cohorts

This study represents 695 subjects from sSA recruited in three different settings, Durban and Kimberley in South Africa, and Gaborone in Botswana. The cohorts are summarized in Table 1, and the entire dataset is available as Supplementary data 1 (doi, 10.6084/m9.figshare.4707316 ^18^). Our study subjects can be summarized according to HIV status as follows:

i *HIV-positive adults and children (n=478):* HIV-positive adults were recruited in sSA through antenatal clinics in both Gaborone (Botswana) and Durban Sinikethemba (South Africa), and mothers and their children attending HIV clinics in Kimberley (South Africa). We have further described chronic viral infections in these groups in previous publications ^19– 22^.
ii *HIV-negative adults and children (n=217):* HIV-negative women were recruited from antenatal clinics in Durban (Masibambisane cohort) and HIV-negative children were recruited via Kimberley Respiratory Cohort (KReC). KReC comprises children aged 9–48 months admitted acutely to the paediatric department at Kimberley Hospital with an admission diagnosis of a respiratory tract infection.

In addition, we used cryopreserved PBMCs from 7 African adults attending HIV outpatient clinics in the UK (Thames Valley Cohort, as previously described ^23, 24^) to screen for *ex vivo* T cell responses (see further details below).

**Table 1.** Cohorts from Botswana and South Africa screened for PARV4, HIV, HBV and HCV.

Cohort location/ name	Study subjects	Number of subjects	HIV status of cohort	Number (%) positive for PARV4 IgG ^a^	Number (%) positive for PARV4 DNA	Number (%) positive for HBsAg ^a^	Number (%) positive for HCV RNA
Gaborone, Botswana	Antenatal women	108	Positive	58/108 (53.7)	0/108 (0)	13/94 (14)	0 (0)
Durban, South Africa; Sinikethemba cohort	Antenatal women	174	Positive	112/174 (64.4)	0/174 (0)	20/172 (11.6)	0 (0)
Durban, South Africa; Masibambisane cohort	Antenatal women	73	Negative	28/73 (38.4)	1/73 (1.4)	6/72 (8.3)	0 (0)
Kimberley HIV cohort, South Africa	Mothers of HIV-positive children	64	Positive	21/43 (48.8)	1/64 (1.6)	4/33 (12.1)	0 (0)
Kimberley HIV cohort, South Africa	Children attending HIV outpatient clinics	132	Positive	24/90 (26.7)	0/132 (0)	0/104 (0)	0 (0)
Kimberley healthy controls, South Africa	Children (HIV-negative siblings of Kimberley HIV cohort)	24	Negative	12/24 (50.0)	0/24 (0)	0/4 (0)	0 (0)
Kimberley Respiratory Cohort, South Africa	Children age 9–48 months admitted to hospital with LRTI	120	Negative (n=117) No data (n=3)	13/120 (10.8)	3/120 (2.5)	1/114 (0.9)	0 (0)
**TOTAL:**		**695**	**Positive: 478** **(68.8)** **Negative: 214** **(30.8)** **No data: 3** **(0.4)**	**268/632** **(42.4)**	**5/695** **(0.7)**	**44/593** **(7.4)**	**0/695** **(0)**

^a^ For PARV4 IgG and HBsAg, the denominator is presented for each group as data were missing for some individuals. LRTI = lower respiratory tract infection

### Ethics approval

Ethics approval was granted as follows: the University of KwaZulu-Natal Biomedical Research Ethics Committee (ref. E028/99), the Health Research Development Committee, Botswana Ministry of Health (ref. PPME-13/18/1); the Oxford Research Ethics Committee and site-specific Research and Development committees (Thames Valley Cohort ref. 06/Q1604/12); Ethics Committee of the Faculty of Health Science, University of Free State, Bloemfontein, South Africa (Kimberley cohorts, ref. ETOVS 08/09 and ECUFS 80/2014). All subjects, or the parent/guardian for children, provided written informed consent for participation.

### Documentation of HIV, PARV4, HCV and HBV status

We screened all 695 subjects for PARV4 DNA and HCV RNA, and had sufficient samples also to screen 632 for PARV4 IgG and 593 for HBsAg.

i **HIV**: HIV-status had been ascertained prior to recruitment and was recorded prospectively. KReC children were deemed to be HIV-negative at the point of presentation to hospital. The majority of these were also screened for HIV infection during their hospital admission episode, with the exception of three children for whom we did not confirm HIV status (these children were included in the HIV-negative group for analysis, based on the clinical data available at the time of admission). HIV-1 RNA viral load was determined by Roche Amplicor Version 1.5 assay (Rotkreuz, Switzerland) or Abbott Laboratories m2000 platform (Abbott Park, IL, USA) (data available for 370/478 HIV-positive individuals). CD4+ T cell counts and percentages were measured by flow cytometry as part of routine clinical diagnostics at the centre of recruitment (data available for 455/478 HIV-positive individuals). High resolution HLA Class I data were also available for 476 HIV-positive subjects, using PCR-sequence specific primer typing, as previously described ^25^.
ii **PARV4 IgG**: we used indirect ELISA, testing 632 samples in duplicate using baculovirus-expressed VP2 and control antigens, as previously described ^4, 11^; arbitrary unit (AU) values were calculated relative to a control sample. Due to a high background reactivity observed in this cohort, we applied an additional stipulation that positive samples must demonstrate a VP2-to-control optical density ratio (ODR) greater than 1.2; samples falling below this cut-off were considered negative.
iii **HBV**: We determined HBsAg status using Biokit enzyme immune assay (Barcelona, Spain) and Murex HBsAg v3 (DiaSorin) assay to detect HBsAg, as previously described ^19^. We were unable to screen the remainder of the cohort for HBsAg due to inadequate sample volumes remaining after other tests had been performed.
iv **HCV:** For HCV detection, we used PCR rather than screening for HCV-Ab, to optimize sensitivity and specificity of the test. RNA was extracted from pooled serum samples (50μl each of 10 samples) using the RNeasy mini kit (Qiagen), according to the manufacturer’s protocol. cDNA was synthesized from 6μl of RNA using Superscript III reverse transcriptase (Life Technologies) with random hexamer primers. PCR reactions were performed using GoTaq DNA polymerase (Promega) and primers listed in Supplementary data 2. First and second round reactions were performed using 2μl of template under the following conditions: initial denaturation at 94°C for 60 seconds and 30 cycles of [18 seconds at 94°C, 21 seconds at 50°C and 60 seconds at 72°C].
To confirm that the lack of detection of HCV by PCR in these samples was not due to a degradation of encapsidated viral RNA, we also screened cDNA samples for a positive control virus. To do this, we screened a total of 575 samples (all samples except KReC cohort) combined into 51 pools, each made up of 10–13 samples (50ul each) using a PCR specific for human pegivirus-1 (HPgV), using previously described methods ^26^. This is sufficiently common in the human population to function as a reliable positive control.
v **PARV4 DNA:** DNA was initially extracted from pooled serum samples (50μl each of 10 samples) using the DNeasy blood and tissue kit (Qiagen), according to the manufacturer’s protocol. For deconvoluted pools and complete genome amplification, 50μl samples were re-extracted individually using the same protocol. PCR reactions were performed using GoTaq DNA polymerase (Promega) and cycling conditions described as above for HCV, using primers listed in Supplementary data 2. Direct amplicon sequencing for PARV4 was performed using BigDye Terminator v3.1 (Applied Biosystems), according to manufacturer’s instructions with both second round primers. Sequencing reactions were read by Edinburgh Genomics (The University of Edinburgh, Edinburgh, Scotland) and assembled using SSE v1.2 ^27^.

### IFN-gamma ELISpot assays

We used cryopreserved PMBCs from 14 subjects who were PARV4 IgG positive, but without PARV4 viraemia (7 children from Kimberley, South Africa, and 7 adults enrolled via the Thames Valley Cohort) to screen for *ex vivo* CD8+ T cell responses using IFN-gamma ELISpot assays. Using methods as previously described ^28^, we quantified IFN-gamma responses to a bank of PARV4 overlapping peptides (OLPs) spanning PARV4 NS, VP and ARF proteins (for peptide sequences see Supplementary data 3, and for a map of the PARV4 proteome, see our previous review ^2^). Subjects and ELISpot data are listed in Supplementary data 4.

Based on responses by HLA-B*5703-positive subjects, we identified a putative epitope within OLPs 9.6 and 9.7. We synthesized three truncations of this epitope (supplied by Schafer-N, Denmark; >80% purity; supplied as lyophilized powders and then dissolved in DMSO) as follows: 8-mer TRITMFQF, 9-mer QTRITMFQF, and 10-mer LQTRITMFQF that most closely matched the binding motif for HLA-B*57:03 (namely A/S/T at position 2 and F/W/Y at the C-terminal position of the epitope) ^29^. Using cells from a PARV4 IgG-positive subject recruited from the Thames Valley Cohort (Patient ID N087), we tested IFN-g ELISpot responses to serial dilutions of these three putative optimal epitope truncations.

### Statistical analysis

GraphPad Software (Prism v.6; http://graphpad.com/) was used for data analysis, using Fisher’s exact test to identify significant relationships between categorical variables, and Mann-Whitney U test for continuous non-parametric data. We used the online logistic regression calculator at Google Sheets ( https://www.google.co.uk/sheets/about/). To investigate whether (i) HLA Class I genotype is predictive of PARV4 IgG status, and (ii) PARV4 IgG status is predictive of either HIV RNA viral load or CD4+ T cell count, we constructed receiver operating characteristic (ROC) curves. As previously described ^17^, our approach was to build predictive models using regularized logistic regression, then estimate the out-of-sample (using 10-fold cross validation) predictive accuracy of the models using ROC curves. This approach allowed us to jointly test for association between all HLA alleles and PARV4 status despite a relatively small cohort.

### Phylogenetic analysis

The evolutionary histories were inferred for PARV4 sequences using maximum likelihood methods implemented using the MEGA 6.0 software package ^30^. The optimum maximum likelihood model (lowest Bayesian information criterion score and typically greatest maximum likelihood value) for the nucleotide sequence alignments was first determined and used for phylogenetic reconstruction. These were the Kimura 2-parameter model with a gamma (γ) distribution for partial VP1 sequences, and the Tamura 3-parameter model with a gamma (γ) distribution for complete NS and complete VP1 sequences.

## Results

### Data visualization

In order to allow visualization of coinfection data subdivided by organism (HBV/HCV/HIV/PARV4), cohort location, sex, and adult/child, we developed a visualization tool using highcharter (A Wrapper for the 'Highcharts' Library. R package version 0.5.0. https://CRAN.R-project.org/package=highcharter Joshua Kunst (2017)). Our visualization can be accessed at the following link: https://purl.oclc.org/coinfection-viz and the code is deposited here: doi, 10.6084/m9.figshare.4750702 ^31^.

### PARV4 IgG prevalence is higher in adults than children

**Figure 1.**
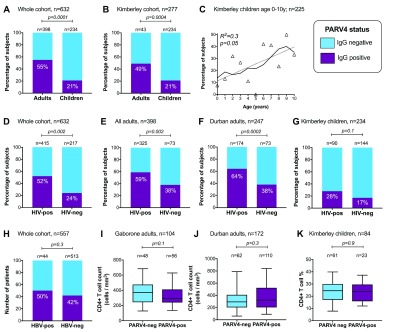
Relationship between PARV4 IgG status and age ( **A**– **C**), HIV status ( **D**– **G**), and HBV status ( **H**– **K**). Boxes show median, 25 ^th^ and 75 ^th^ centiles; whiskers show 5 ^th^–95 ^th^ centiles. P-values by Fisher’s exact test (bar charts), linear regression (scatter plot), and Mann Whitney U test (box and whisker plots). Denominator stated on each panel varies based on availability of relevant data.

Overall, PARV4 IgG prevalence in this study was 268/632 (43%). Table 1 shows the breakdown of seroprevalence by cohort. Consistent with our previous findings ^11^, adults were significantly more likely to be seropositive than children (238/492 (48%) in adults vs. 50/234 (21%) in children; p<0.0001; Figure 1A). We also observed this relationship within the Kimberley cohort (22/43 adults vs. 50/234 children; p<0.0001; Figure 1B). Among children age 0–10 years, there was a trend towards an increase in PARV4 seroprevalence over time ( Figure 1C).

### Co-infection analysis: PARV4 IgG is associated with HIV, but not with HBV or HCV

We identified a significant association between PARV4-IgG status and HIV infection (p=0.002; Figure 1D). This relationship also holds among adults, and in the single setting of Durban (p=0.002, Figure 1E; p=0.0002, Figure 1F, respectively). Similarly, in children there was a trend towards higher PARV4-IgG positivity in the context of HIV infection, although this did not reach statistical significance (p=0.1; Figure 1G). PARV4 and HBV infection were not statistically associated among 557 patients (p=0.3; Figure 1H). No subject in this study was positive for HCV RNA. However, 17 out of the 51 sample pools were found to be positive for our control virus, human pegivirus-1, suggesting an overall prevalence comparable to previous reports ^32^ and supporting a true absence of HCV viraemia.

Among HIV-infected adults, there was no significant relationship between PARV4 IgG status and CD4+ T cell count in Gaborone or Durban ( Figures 1I and J, respectively), and in children there was no relationship between PARV4 IgG and CD4+ percentage ( Figure 1K). There was also no relationship between PARV4 and HIV viral load (data not visualised). On logistic regression analysis of 557 subjects for whom we held a complete dataset (data available for all variables), PARV4 IgG status remained associated with HIV status (p<0.0001), but no relationship was seen with sex, cohort location, adult/child or HBsAg status.

### Lack of association between HLA Class I genotype and PARV4 IgG status

Given the established protective role of certain HLA alleles or loci in control and clearance of viral infection in previous studies of these populations ^17, 20, 21^, we sought any evidence for a relationship between HLA Class I genotype and PARV4 serostatus among HIV-positive individuals. We found no such association, either using the entire class I genotype (ROC Area Under the Curve [AUC]=0.62, compared to AUC=0.60 when only cohort labels were used as predictors; p=0.18 against null model that AUC is greater when including HLA alleles as predictors), or analyzing independently by class I locus (AUC=0.62, 0.62, and 0.58, for HLA-A, HLA-B and HLA-C, respectively; p>0.1 for all comparisons).

### PARV4 sequences from South Africa cluster with Genotype 3 sequences from Cote d’Ivoire

To investigate the prevalence of PARV4 viraemia, with a particular interest in exploring the idea that reactivation of latent virus may occur in the variable states of immunocompromise associated with HIV or pregnancy, we screened this composite cohort for evidence of PARV4 viraemia using a previously described tetraparvovirus PCR ^33^. We identified five viraemic subjects among our cohort of 695 (0.7%): three HIV-negative children from Kimberley (KReC009, KReC089 and KReC102), one HIV-positive child from Kimberley (K172C) and one HIV-negative antenatal woman from Durban (Masi039).

**Figure 2.**
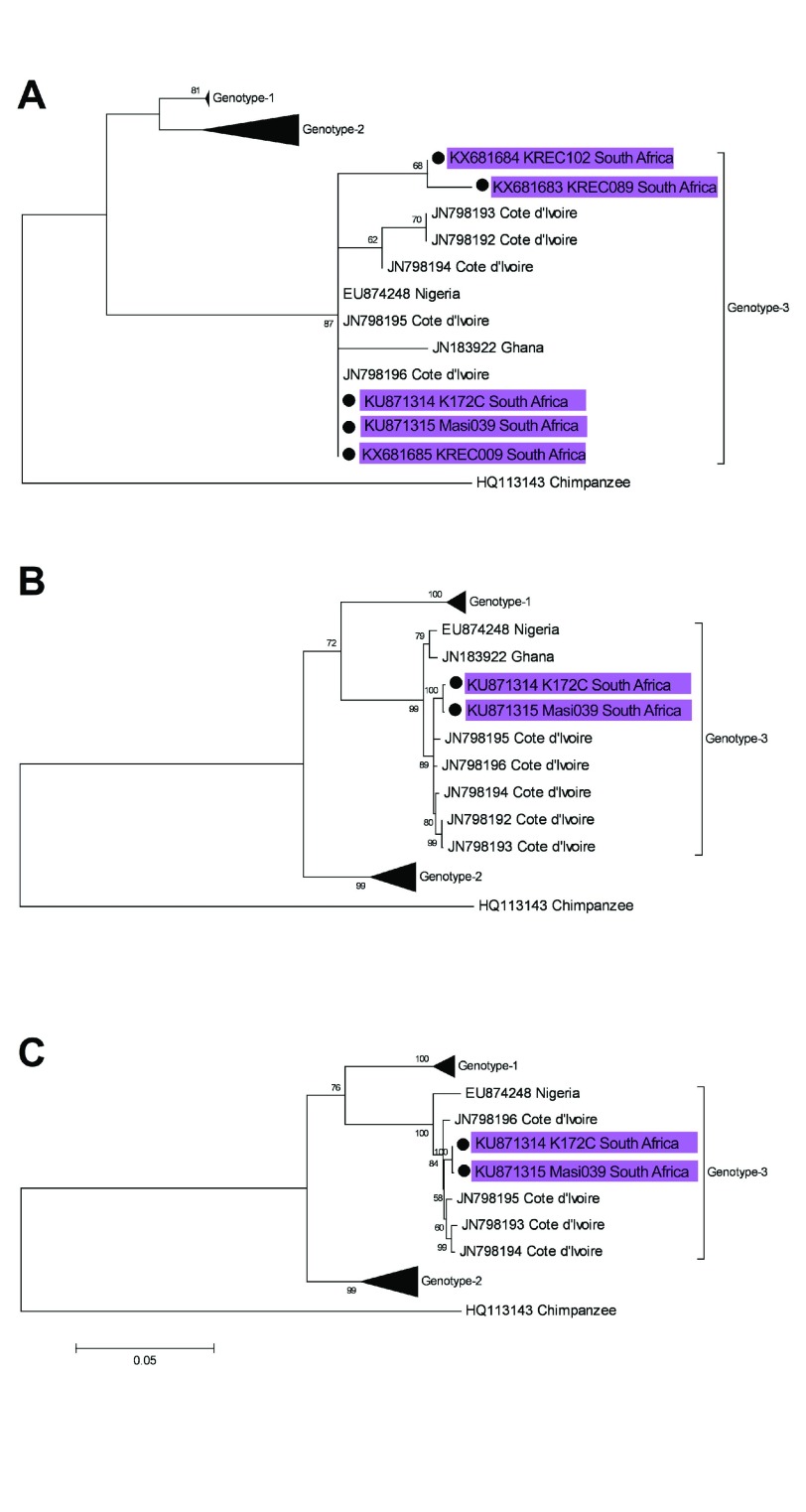
Phylogeny of PARV4 sequences detected in serum from five individuals from South Africa. Phylogeny inferred using maximum likelihood trees from partial VP1 ( **A**), complete NS ( **B**) and complete VP1 ( **C**) nucleotide sequences (equivalent to nucleotides 3067-3310, 283-2271 and 2378-5035, respectively, of the PARV4 reference sequence NC007018). In each case, the new sequences derived from South Africa are highlighted (lavender bars). Bootstrap support of branches (500 replications) is indicated.

Phylogenetic analysis of the tetraparvovirus PCR amplicons from the five viraemic individuals revealed that all were genotype-3 ( Figure 2A). From two individuals, K172C and Masi039, we generated complete viral genome sequences using overlapping PCR; the fully assembled sequences have been submitted to GenBank (accession numbers KU871314 and KU871315). For the remaining individuals, only a subset of overlapping genome PCR reactions was positive (one or two of the seven reactions), so we were unable to assemble a full genome sequence. This suggests a low titre of virus in these individuals and further repeat reactions could not be performed due to limited sample volume. However, we have submitted the partial VP1 sequences used for the phylogenetic analysis of these three individuals to GenBank (accession numbers KX681683, KX681684 and KX681685).

The K172C and Masi039 sequences show a high degree of similarity to each other showing >99% nucleotide identity across the genome. Phylogenetic analysis of the complete NS and VP1 coding regions ( Figures 2B and C) again demonstrates a clear grouping with previously reported PARV4-genotype 3 sequences, particularly those obtained from individuals in Cote d’Ivoire ^34^.

### High breadth and magnitude of CD8+ T cell responses to PARV4

**Figure 3.**
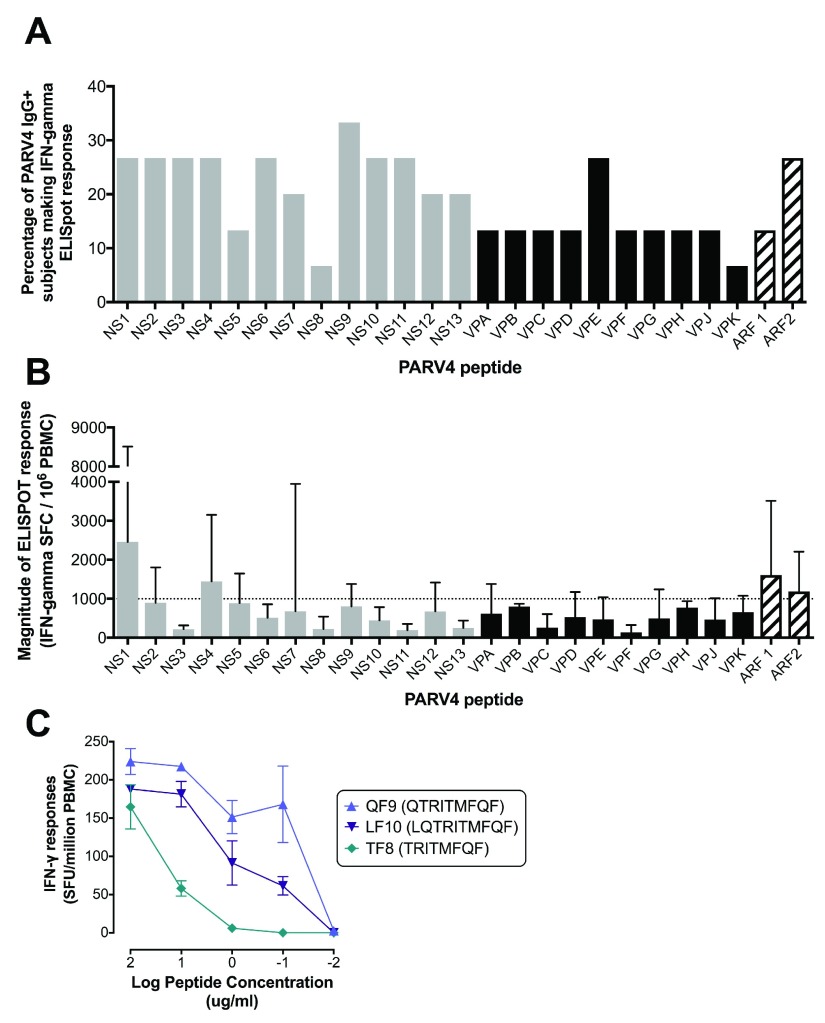
IFN-gamma CD8+ T cell responses to PARV4 peptides determined by ELISpot assays. Data in ( **A**) and ( **B**) are derived by screening 14 subjects positive for PARV4 IgG recruited from the Kimberley (n=7, children) and Thames Valley (n=7, adults) cohorts. None of these subjects was PCR positive for PARV4 from serum. Raw data showing the responses made by each individual subject can be viewed in Supplementary data 4. ( **A**) Proportion of 14 screened subjects who made each individual response. ( **B**) Mean magnitude (box) and range of response (whiskers); the dashed horizontal line allows visualization of peptides for which the mean response is >1000 SFCs/10 ^6^ PBMC. Responses to NS peptides are shown in grey, to VP peptides in black, and to ARF in hatched bars. ( **C**) Prediction of a novel HLA-B*5703-restricted CD8+ T cell epitope in PARV4 NS protein. Cryopreserved PBMCs from PARV4 IgG-positive adult subject N087 (HIV-positive adult recruited via the Thames Valley cohort, HLA class I genotype HLA-A*0301/-A*3001/-B*5703/-B*5801/-C*0602/-C*1801) were screened by IFN-gamma ELISpot for responses to peptide truncations from PARV4 NS protein (sequences within OLPs 9.6 and 9.7) at different concentrations. Plots and error bars show mean and SEM of assays performed in triplicate. On the basis of the HLA-B*5703 binding motif and the greatest magnitude responses, the putative optimal epitope is HLA-B*5703-QF9 (QTRITMFQF).

Among 14 individuals screened for *ex vivo* CD8+ T cell responses, we demonstrated IFN-gamma ELISpot responses to peptides spanning all three PARV4 proteins ( Figure 3A), including high magnitude responses (mean response >1000 spot forming cells/10 ^6^ PBMCs) to NS1, NS4, ARF1 and ARF2 ( Figure 3B). Children made a median of 5 responses (range 1–12), while adults made fewer responses (median 3, range 1–5), but this difference did not reach statistical significance (p=0.12, Mann Whitney U test; data not visualised). We tested one predicted optimal epitope using three possible peptide truncations found within OLPs 9.6 and 9.7, confirming that the peptide QF9 (QTRITMFQF) found within PARV4 NS protein is the most likely HLA-B*57:03 restricted epitope ( Figure 3C).

## Discussion

### Epidemiology of PARV4 and HIV, HBV and HCV mono-infection and co-infection

In keeping with previous studies of sSA, we report a PARV4 IgG seroprevalence that is strikingly higher than in Western Europe. In this setting, we conclude that there is no evidence that HBV or HCV infection is associated with PARV4 in sSA. The HBsAg data reported here are broadly in keeping with previous epidemiological studies of southern Africa ^35^; however, ongoing surveillance will be required in these populations to determine the changing prevalence of infection following more widespread introduction of the prophylactic HBV vaccination in infancy ^22, 36^. The lack of HCV in these cohorts is of interest and in striking contrast to high rates of HBV. Antibody screening for HCV can be problematic, both because of reported concerns regarding false positive tests, and because of the problem in discriminating between active infection and previous cleared infection ^37, 38^. We therefore aim to have increased both the sensitivity and specificity of testing by using a molecular test for HCV.

Here we have shown a significant relationship between HIV infection and PARV4 serostatus in adults. A previous analysis of a smaller cohort reported an unexpected negative correlation between PARV4 IgG and HIV status in children ^11^, but no such effect was seen in this expanded cohort; it is plausible that the previous effect was confounded by another factor (of which age is the most likely). The reasons for the difference in seroprevalence and associations with BBVs remain uncertain, but in this case may relate to increased susceptibility to PARV4 infection in the setting of reduced cell-mediated immunity mediated by HIV infection, or may relate to characteristics or behaviours of the host population, environmental factors, viral genetics, or a combination of these factors ^14^.

In the three settings studied here, there is no evidence that PARV4 serostatus is associated with HIV progression, either in adults or children. A previous paper that reports an association between positive PARV4 IgG status and more advanced HIV disease acknowledges the potential confounding influence of co-infecting HCV in PARV4-positive individuals ^39^.

### PARV4 viraemia and phylogeny

Even in this cohort from an endemic region, enriched for both pregnancy and HIV infection, we were able to amplify PARV4 DNA from only <1% of all individuals screened. Four of the five viraemic individuals were children, who may be more likely to be experiencing a primary infection. Interestingly, three of the subjects with low-grade viraemia were KReC (Kimberley Respiratory Cohort) children, concordant with the hypotheses either that PARV4 infection might cause or contribute to respiratory illness in young children, or that respiratory tract infections make children more vulnerable to primary PARV4 infection or to low-grade reactivation of viraemia. This association has been previously postulated ^12^, but not further explored. Future careful studies, enrolling large numbers of study subjects and collecting detailed prospective diagnostic data would be required to expand on this investigation.

Although our approach to detecting viraemia represents only a cross-sectional ‘snap shot’, these data suggest that acute infections are relatively short-lived, and that subsequent immune containment is generally successful. Overall, therefore, these data do not support the hypothesis that either vertical or blood-borne transmission is likely to be highly epidemiologically significant in driving the high PARV4 seroprevalence in sSA.

We confirmed the identity of the circulating viruses as genotype-3, in keeping with other sequences from Africa ^40^. Sequence differences between genotypes could potentially contribute to a phenotype difference that accounts for the differing transmission and prevalence of PARV4 between Africa and Europe; more work is needed to elucidate the biological effect of sequence differences between genotypes.

### CD8+ T cell responses

Although viraemia was uncommon, we found CD8+ T cell responses to PARV4 spanning the entire viral proteome, and of particularly high magnitude in certain regions of NS and ARF proteins. These responses, in the absence of detectable viraemia, support the view that PARV4 may behave similarly to chronic herpes viruses, particularly CMV, in which a latent reservoir underpins episodic reactivation, maintaining T cell responses in the long term ^41^. Previous reports quantifying the CD8+ T cell response using the same *in vitro* approach have focused on NS peptides as being immunodominant ^5, 7^. These current data therefore represent new evidence for significant CD8+ T cell responses to both VP and ARF proteins, with particularly striking high magnitude responses to ARF.

### Caveats and limitations

The amalgamation of subjects recruited within pre-existing cohorts has allowed us to make some new advances in a manner that is cost and time-effective, but this leaves certain important questions unanswered. This work is limited in being a retrospective approach, by the limited and variable demographic characterization of the cohorts, and by the potential confounding factors in operation. In particular, differences observed between cohorts may be related to factors such as age and sex of study participants.

## Conclusions

In summary, these data represent an advancement of our understanding of PARV4 in sSA, mainly by permitting us to study a larger cohort than has previously been amalgamated in this setting. However, much remains to be elucidated about the epidemiology (specifically in understanding routes of transmission and differences between geographical settings), as well as an ongoing need to determine the clinical significance of this virus. These questions are likely to be particularly important for African populations in which PARV4 is so highly endemic.

## Data availability

Supporting metadata and experimental reagents for this study are available on Figshare, DOI 10.6084/m9.figshare.4707316 ^18^, which contains the following files:

**Supplementary data 1:** Cohort data for 695 study participants enrolled in Durban, Kimberley and Gaborone.

**Supplementary data 2:** PCR primer sets for human parvovirus 4 (PARV4) complete genome sequencing and hepatitis C virus (HCV).

**Supplementary data 3:** Overlapping peptide set used for screening PBMCs from PARV4 IgG positive subjects using IFN-gamma ELISpot assays.

**Supplementary data 4:** Results of screening 14 PARV4 IgG positive individuals for IFN-gamma ELISpot responses to overlapping peptides spanning the entire PARV4 proteome.
