# Supplementary material for: PARV4 prevalence, phylogeny, immunology and coinfection with HIV, HBV and HCV in a multicentre African cohort
Source: Wellcome Open Res. 2017 Apr 7;2:26. [Version 1] doi: 10.12688/wellcomeopenres.11135.1 (PMC5423528; doi:10.12688/wellcomeopenres.11135.1)
Supplement: Peer review history

# Peer review: Referee response for version 1

**DOI:** 10.21956/wellcomeopenres.12013.r22013

**Published:** 2017-05-08

**Authors:** Klaus Hedman

**Affiliations:** 1 Department of Virology, University of Helsinki, Helsinki, Finland

The manuscript by Sharp et al. confirms and extends our knowledge on the epidemiology and T-cell immunity of an emerging human virus, in a comprehensive sub-Saharan cohort. The methods, data and conclusions appear on the whole appropriate. I have only one major and a few few minor questions/comments for the authors' consideration.

 Major

1. Page 5, para "Co-infection analysis: PARV4 is associated..." In complete absence of positive HCV findings in this manuscript, albeit by PCR, it does not feel appropriate to conclude (here or elsewhere in this manuscript, e.g. Discussion) that "PARV4 IgG is associated...not with...HCV." We cannot comment on an association or its absence with a counterpart that did not occur in the material studied, can we?

Minor

1. Page 3, para "In addition, we used cryopreserved PBMCs from 7 African adults...".
  The authors might want to mention here also the 7 children that were studied (p. 5); and the corresponding PARV4 statuses?
2. Page 3, last para, "KReC", please spell out, perhaps also in Abbreviations.
3. Page 5, para "To confirm that the lack of detection of HCV by PCR..." As a matter of fact, Ref. 26  concludes: "...active infections with HHpgV-1 were infrequently detected in blood, even in groups that had substantial parenteral exposure." Does such a virus qualify for a positive control? Or have higher prevalences been observed in other papers that could be cited here?
4. Page 10 second para, "suggest that acute infections" - do the authors mean "acute" or "active" (or "active primary")?

I have read this submission. I believe that I have an appropriate level of expertise to confirm that it is of an acceptable scientific standard.

# Peer review: Referee response for version 1

**DOI:** 10.21956/wellcomeopenres.12013.r22113

**Published:** 2017-04-24

**Authors:** Eric Delwart

**Affiliations:** 1 Blood Systems Research Institute, San Francisco, CA, USA

The manuscript by Sharp et al. describes the relationship between sero-positivity to a human parvovirus (PARV4) and the detection of anti HIV antibodies, HIV viral loads, HBsAg detection and HCV RNA in a large number of children and adults from sub-Saharan Africa (sSA). As previously reported in sSA a large fraction of tested samples (42%) were sero-positive for PARV4 with a statistically greater number of those also being HIV sero-positive rather than HIV sero-negative. Adults showed a greater rate of PARV4 antibody detection than children. No HCV RNA was detected and there was no correlation between PARV4 antibody detection and presence of HBsAg.

 This is a nicely performed study also showing that children are being infected by a still unknown route (4/5 PARV4 DNA positive samples were from children and children showed antibody positivity rate of 21%). The higher rate of PARV4 antibodies in HIV infected individuals is reminiscent of the situation in Europe/US where PARV4 infection focused in adults with HIV and/or HCV infections and heamophiliacs, presumably through injection drug use or other forms of blood and blood products contacts. The current studies also confirms the predominance of PARV4 genotype 3 in sSA while European and North American are mostly infected with genotypes 1 and 2.

 Whether PARV4 is acquired through the same routes as HIV seems likely although how children become infected remains unclear. Infection in children may occur through the usual routes for parvoviruses which includes both the respiratory track (human parvovirus B19) or the fecal oral route (canine parvovirus). Unlike the situation in adults the rate of PARV4 antibody detection in children was not higher in HIV positive children.

 No association was found between PARV4 antibodies and either CD4 counts and HIV viral load. The persistent high level of CD8 T cell response to PARV4 peptides seems to indicate the lingering presence of antigens despite low rate of detectable viremia (5/695). This is interpreted as reflecting occasional reactivation from latent reservoirs although replication below the level of detection in the still unknown cellular target of PARV4 could also account for the strong CD8 responses.  

 Last word abstract could replace “…clinical significance of this organism”. by “…clinical significance of this recently identified virus”.

 Page 6 it is mentioned that there is no relationship PARV4 antibodies with sex, cohorts, location, adult/child or HBsAg status. Isn’t the statement about adult/child contradicted by claim in second paragraph of results that adults have greater rates of PARV4 antibodies than children?

 In discussion insert reference about sero-prevalence being strikingly higher than in Western Europe.

 The following sentence could use more clarity: Overall, therefore, these data do not support the hypothesis that either vertical or blood-borne transmission is likely to be highly epidemiologically significant in driving the high PARV4 seroprevalence in sSA.

 Couldn’t the high rate of PARV4 IgG in children reflect at least in part vertical infection from infected mothers? Couldn’t blood borne infection account for the association with HIV infection acquired through injection drug use? Is the lack of association with HBsAg used to infer that PARV4 not transmitted through injection drug use? That paragraph could be expanded. Couldn’t PARV4 be transmitted by both the oral route in children and by blood contact in adults much like parvovirus B19?

 PARV4 is compared to CMV but could also be compared to parvovirus B19 whose DNA has been detected in skin tissues for years after infection.

I have read this submission. I believe that I have an appropriate level of expertise to confirm that it is of an acceptable scientific standard.
